# The Book World of Medicine and Science

**Published:** 1893-12-02

**Authors:** 


### THE BOOK WORLD OF MEDICINE AND SCIENCE.

A GUIDE TO THE EXAMINATION OF THE URINE. By J. WICKHAM LEGG. Edited and Revised by H. LEWIS JONES, M.A., M.D. (Seventh edition, 1893. Fcap. 8vo., pp. 139, price 3s. 6d.)

By the time a book has reached its seventh edition it is clear that it meets a general demand, and that it satisfies its readers; therefore the reviewer's task is easy. In spite, however, of the popularity of this book, we must confess to some disappointment in the seventh edition of it. The medical student is required now to be so much more exact in his knowledge than he was when the first edition of this book appeared that we wish Dr. Lewis Jones had made the present volume a little more precise. For instance, on page 5 we might have been told that the colour of the urine of patients taking santonin is not the same when the urine is acid as when it is alkaline; also as carboluria is an important symptom the tint of the urine might have been better described than by the word dark, which might equally mean dark red or dark blue. On the same page, too, in speaking of the causes of pale urine, no mention is made of chronic Bright's disease. Then again, on the next page, it is stated that certain drugs, as copaiba and sandal-wood oil, give a characteristic smell to the urine. Surely it would have been more accurate to state precisely what the drugs are. On page 13 we notice that the only causes given for a high specific gravity of the urine is glycosuria or an excess of urea. We had always thought that there were other causes besides these two. It would have been valuable for the student if on page 16 it had been pointed out that there are other substances than benzoic and, as for example, salicylic acid which increase the acidity of the urine; and if on page 22 more details had been given of the precipitate caused by the addition of nitric acid to the urine of those taking copaiba. Then again, we ought to have had a fuller account of the substances other than sugar which will reduce Fehling's solution.

The latter part of the book treats of the quantitative estimation of urinary constituents. For the ordinary medical student too much attention is here given to the polariscope. We hardly think it quite fair to state that no easy, rapid, and trustworthy method of estimating albumen has yet been suggested. The chief requisite is that the test should enable us to compare the amounts of albumen on different days, and although Esbeck's tubes are not quite accurate, they are sufficiently so for comparative results. There are several other points to which we might draw attention, but perhaps we have mentioned enough. We have not alluded to them in any unfriendly spirit, but only in the hope that in the eighth edition the book may be still further improved.

THE PHILOSOPHY OF CRIME: A Paper read at the International Congress of Charities, Correction, and Philanthropy, Chicago, June 12th to 17th, 1893. By C. H. REEVE, of Plymouth, Indiana. (Plymouth, Ind.: D. M'Donald and Co.)

"What is crime? It is the manifestation by a human being of a mental impulse in some act which has been forbidden by the declaration of other human beings, in pursuance of certain human customs called government." With this dictum Mr. Reeve begins his paper. On such a principle any absolute definition of crime is impossible, nor does he

THE BOOK WORLD OF MEDICINE AND SCIENCE—*continued.*

attempt it. The same acts are not held in the same esteem in all countries, nor in all ages. In classic times suicide was held to be an honourable death; now we punish those who unsuccessfully attempt it in order to deter others from trying to take their own life. Moreover, circumstances alter cases. It is criminal to kill a man, but we do not arraign a successful general as a murderer. Even in those acts which are generally acknowledged to be criminal, circumstances of environment, provocation, and temperament intervene to modify the degree of guilt attaching to them. Nevertheless, though it is impossible to give a definition of crime as accurate as the elucidation of a problem in Euclid, the general common sense of mankind in civilised lands gives a sufficient statement of its nature for practical purposes.

Without the law there is no sin. Without some degree of civilisation there is no crime. The savage takes what he wants and slays whoever would prevent him with no sense of guilt. It is only when higher views come in, notions of interdependence, of communal or tribal rights and welfare, that those whose action is opposed to that of the community become criminals. This may be held to mitigate in some degree the criminal's responsibility; but in reality we have attained such a degree of self-consciousness that the dullest brute who lifts his hand to steal or strike knows that he is injuring others for his own advantage, supposed or real. He knows it, but, knowing, can he restrain himself? This is the point in which the physiologists and the moralists are at issue; but the discussion is of only academic interest, for both are agreed that whether the criminal cannot, or merely will not, cease from sinning, society has the right to prevent him from wronging his fellows. And it is the physiologists who would take the most drastic measures for the repression of crime.

Mr. Reeve is one of the physiologists. "Every criminal impulse," he says, "is evidence of an abnormal condition of the mental organism. It may be a result more or less of an abnormal condition of the physical organism." But he does not, therefore, bid the criminal go free. "Whatever the cause or condition, the person is a menace to order, to government, and so to liberty. . . . Take the dangerous subject into custody, and put him in a place and condition where he will not be dangerous." This imprisonment is not to be the brief incarceration which is common; every sentence is to be for life, though it may be modified if the "abnormal condition" which produced the criminal impulse can be removed. But if not, there is to be no commutation. There is a strong reason for recommending a course apparently so severe. Mr. Reeve has noticed, with the same dismay as other observers, that the criminal classes are increasing out of due proportion to the rest of the population. Neither the penalties of the law, the spread of education, nor the efforts of religious bodies avail against the increasing tide of crime. Therefore, it seems that some new method must be adopted. Mr. Reeve's intention in thus shutting up the criminal is similar to that which justifies society in putting the lunatic in an asylum. He must be kept from committing further mischief himself, he must at all hazards be kept from perpetuating his evil propensities in a criminal posterity. Mr. Reeve goes so far as to recommend assexualisation in cases of a "confirmed criminal diathesis," so that, even if he should escape from custody, his power for evil may be limited to himself. It will be some time before such an idea as this will be generally entertained. It is entirely opposed to the popular idea of reforming the criminal, and will certainly have to endure much harsh criticism. But it is impossible not to appreciate the spirit in which it is put forth, and the thought and earnestness which mark Mr. Reeve's little pamphlet.

